# Provider performance in treating poor patients - *factors influencing prescribing practices in lao PDR: a cross-sectional study*

**DOI:** 10.1186/1472-6963-11-3

**Published:** 2011-01-06

**Authors:** Lamphone Syhakhang, Douangdao Soukaloun, Göran Tomson, Max Petzold, Clas Rehnberg, Rolf Wahlström

**Affiliations:** 1 Food and Drug Department, Ministry of Health, Vientiane, Lao PDR; 2 Mahosot Hospital, Ministry of Health, Vientiane, Lao PDR; 3 Division of Global Health (IHCAR), Department of Public Health Sciences, Karolinska Institutet, Stockholm, Sweden; 4 Medical Management Centre (MMC), Karolinska Institutet, Stockholm, Sweden; 5 Nordic School of Public Health, Göteborg, Sweden; 6 Family Medicine and Clinical Epidemiology, Department of Public Health and Care Sciences, Uppsala University, Uppsala, Sweden

## Abstract

**Background:**

Out-of-pocket payments make up about 80% of medical care spending at hospitals in Laos, thereby putting poor households at risk of catastrophic health expenditure. Social security schemes in the form of community-based health insurance and health equity funds have been introduced in some parts of the country. Drug and Therapeutics Committees (DTCs) have been established to ensure rational use of drugs and improve quality of care. The objective was to assess the appropriateness and expenditure for treatment for poor patients by health care providers at hospitals in three selected provinces of Laos and to explore associated factors.

**Methods:**

Cross-sectional study using four tracer conditions. Structured interviews with 828 in-patients at twelve provincial and district hospitals on the subject of insurance protection, income and expenditures for treatment, including informal payment. Evaluation of each patient's medical record for appropriateness of drug use using a checklist of treatment guidelines (maximum score = 10).

**Results:**

No significant difference in appropriateness of care for patients at different income levels, but higher expenditures for patients with the highest income level. The score for appropriate drug use in insured patients was significantly higher than uninsured patients (5.9 vs. 4.9), and the length of stay in days significantly shorter (2.7 vs. 3.7). Insured patients paid significantly less than uninsured patients, both for medicines (USD 14.8 vs. 43.9) and diagnostic tests (USD 5.9 vs. 9.2). On the contrary the score for appropriateness of drug use in patients making informal payments was significantly lower than patients not making informal payments (3.5 vs. 5.1), and the length of stay significantly longer (6.8 vs. 3.2), while expenditures were significantly higher both for medicines (USD 124.5 vs. 28.8) and diagnostic tests (USD 14.1 vs. 7.7).

**Conclusions:**

The lower expenditure for insured patients can help reduce the number of households experiencing catastrophic health expenditure. The positive effects of insurance schemes on expenditure and appropriate use of medicines may be associated with the long-term effects of promoting rational use of drugs, including support to active DTC work.

## Background

Doctors' prescribing practices have considerable bearing on the effectiveness of health care treatments. There are many factors influencing prescribing, e.g., existing laws and regulations, types of patients, different insurance schemes, informal payment, drug promotion, medical product representatives and other incentives [1-5]. Therefore, questions concerning appropriateness of treatment are of interest among health care professionals, policy-makers, administrators and researchers. Informal payment, i.e. patients' decision to pay extra to the health care provider, in money or in kind, has been listed as one problem in many health systems [6,7].

In Lao People's Democratic Republic (Laos) the functioning of health services in hospitals at different levels has improved gradually over the last ten years, especially regarding rational use of medicines [8,9]. Drug and Therapeutic Committees (DTC), and standard treatment guidelines (STGs) to develop quality of care have been established at all provincial and district hospitals, including remote and rural areas [10].

Four main types of insurance schemes are currently being piloted in selected provinces in Laos: (1) the social security system covering civil servants (CSS), (2) health insurance schemes for the private sector (SSO), (3) community-based health insurance schemes (CBHI), and (4) health equity funds (HEF) for the poor. The payment under CSS is the responsibility of the government, whereas the SSO is paid by the private companies, and CBHI gets contributions directly from the households. The Swiss Red Cross has supported HEF in Vientiane province and Namebak district in Luangprabang province. For all schemes, payments to contracted hospitals are on a capitation basis and limited to a defined set of services, with the exception of HEF.

An exemption policy for poor people was launched in Laos in 1995, but has not been effective due to a limited government budget and an unclear definition of the term "poor people". About 60% of health care expenditures come from individuals' own pockets [8]. Increasing out-of-pocket expenditures in public and private health care services are driving many families into poverty, and are further burdening those who are already poor [11]. In addition, in order to gain access to public hospitals and to receive a higher quality of services, in some countries, informal payments are widespread and a major source of inequity and inefficiency in health care systems [6,7].

Poverty and illness constitute a vicious circle, especially in low- and middle-income countries [12], including Laos. Low quality of services and inappropriate practices of health providers, especially in contact with poor patients, may result in ineffective health care, unnecessary expenditure and a negative impact on patients' health and finances. Many studies on public health facility services have been carried out in Laos, however no study has been performed on provider behaviour in relation to poverty and illness.

The main objectives of this study were: 1) to assess the appropriateness of treatment by health care providers in the encounter (a) with poor patients, (b) patients with and without insurance; 2) to determine the expenditure for treatment of different types of patients; and 3) to assess factors associated with provider performance.

## Methods

### Study setting

This cross-sectional study was conducted in hospitals in three provinces in the north, middle and south of Laos, Luangprabang, Vientiane and Savannakhet, where different insurance schemes have recently been piloted. Three districts in each province were purposively selected to include districts where one or two of the available security systems, CBHI and HEF, had been introduced: Namebak, Ngoy and Xiengngeuane districts in the Lauangprabang province; Vangvieng, Phone Hong and Xanakham in the Vientiane province; and Champhone, Outhoumphone and Sepone in the Savannakhet province.

### Sample size and sampling procedures

The provincial hospital and the three district hospitals in each province were included - a total of 12 public health facilities. One of the district hospitals in each province was involved in the insurance scheme pilot, while the other two operated without any insurance scheme. Four tracer conditions were used for data collection: a) major injury (including bone fracture), b) hypertension, c) post-delivery haemorrhage or Caesarean section, and d) pneumonia in children under five. Patients were selected consecutively according to the time of their admission to the hospital. The aim was to recruit 20 patients per disease and per health facility, but in spite of a period of five months for data collection we could only include a total of 828 patients.

### Data collection methods

Data were collected through structured interviews with in-patients on the surgical, medicine, obstetric and paediatric wards. Data from the same patients' medical records were also collected. For patients with hypertension, data were mostly collected in out-patient wards.

The interviews with patients used a structured questionnaire specifically developed for the purpose of this study. The questionnaire was pretested and modified before use. The questions covered reasons for choosing the facility, opinion about the service received and the quality of care, information about service fees, including informal payment, general satisfaction and expectations, and recommendations for better health services. The questionnaire was not finalised until the day of discharge to ensure that all expenditure were recorded. Demographic data and information about household income were also collected via the questionnaire.

Information about the use of medicines and diagnostic tests was retrieved from the patient records, including the expenditure for medicines and diagnostic tests, fees and other expenditure. The expenditure for prescribed medicines and diagnostic tests were based on the price lists compiled by the pharmacy unit at each hospital. Fees were usually quite small and relate to charges for consultation and documents. Other expenditure refer to all other expenditure for the treatment episode, including cost for equipment, transportation, accommodation and food for patients themselves and their relatives. The questions on informal payment addressed whether the patients had contributed any additional payment or donated food or gifts of any kind to any of the health care providers.

The appropriateness of use of medicines was assessed by using a checklist and a scoring system, which was based on treatment guidelines used in the hospitals for the four tracers. Correct use of medicines according to the guidelines, including intravenous fluids and blood transfusions, received a positive score, while inappropriate medications received negative scores. The maximum score was 10 for all tracers.

Household income was classified into three levels related to the specific poverty line measurement of 100 000 LAK (11 USD) per individual per month, as indicated in the Lao Expenditure and Consumption Survey (LECS II). This amount corresponds to the money required to purchase 2 100 calories of food per person per day, plus a non-food allowance [13,14].

Data were collected from October 2007 to March 2008 by trained data collectors with the supervision of the principal investigator (LS) and the research team, which visited all hospitals.

### Data analysis

The standard for each tracer condition and the corresponding checklists were developed by specialists from the main central hospital, and used for evaluation of the appropriateness of treatment and use of medicines.

Epi info version 6.4 and Stata 10 were used for data input and analysis. Frequencies, cross-tabulations, means and standard deviation have been performed. P-value was used to compare the prescribers' performance between different types of patients. Multiple linear regression analysis was performed to identify factors influencing higher expenditure for treatment.

Ethical clearance was obtained from the National Ethical Committee for Health Research, Ministry of Health, Laos. All participants were informed about the study purpose and gave verbal consent after explanation that all collected information would be handled anonymously.

## Results

There were in total 828 in-patients (45% women) in the three provinces. Of these, 201 had suffered a major injury (7 were insured), 203 had hypertension (63 insured), 201 had suffered a post-delivery haemorrhage or had undergone a Caesarean section (25 insured) and 223 were children under five with pneumonia (21 insured). The average income per family member per month was LAK150 000 (US$18), ranging from LAK 4 000 to LAK 4 000 000 (Table 1). The demographic characteristics are in concordance with information gathered from national surveys [14].

**Table 1:** Patients background and characteristics

Description	Frequency N = 828	%
**Tracer conditions**		
1. Major injury or bone fracture	201	24
2. Hypertension	203	25
3. Post-delivery haemorrhage or Caesarean section	201	24
4. Pneumonia in children under five	223	27
**Sex**		
Female	375	45
Male	453	55
**Professions**		
1. Farmer	258	31
2. Civil servant	359	43
3. Private sector employee	124	15
4. Student	52	6.3
5. Others	27	3.3
**Education**		
0. None	146	18
1. Primary school	266	32
2. Lower-secondary school	205	25
3. Upper-secondary school	174	21
4. University	15	1.8
**Income/month**		
Median (min-max)	150 000 (4 000-4 000 000)	
Income level 1: ≤100 000 LAK	291	36
Income level 2:101 000-200 000 LAK	217	27
Income level 3: ≥201 000 LAK	310	38

The score for appropriate drug use was significantly higher for insured patients than patients without insurance. Insured patients received significantly fewer medicines in total and also fewer antibiotics compared with uninsured patients. Insured patients also stayed fewer days in hospital and paid significantly less for medicines and diagnostic tests than uninsured patients (Table 2).

**Table 2:** Use of medicines, diagnostic tests and costs in LAK* for uninsured and insured patients

Description	Insurance Mean (SD)		p-value
		
	No N = 712	Yes N=116	
Appropriate drug use score	4.9(2.7)	5.9(2.7)	0.0001
Total number of medicines during hospital stay	5.4(2.5)	5.2(3.2)	0.04
- Number of antibiotic injections	0.98(0.89)	0.52(0.86)	< 0.0001
- Number of oral antibiotics	0.49(0.58)	0.37(0.68)	0.011
- Number of essential medicines	4.6(2.1)	4.6(2.6)	0.14
- Number of brand drugs	1.3(1.4)	1.2(1.3)	0.54
Average duration of stay (days)	3.7(4.4)	2.7(3.3)	0.002
Cost of medicines (in LAK)	373 060 (772 660)	126 063 (267 340)	0.001
Cost of diagnostic tests (in LAK)	77 826 (123 104)	50 146 (54 929)	0.02
Other costs (in LAK)	360 608 (563 086)	240 745 (394 973)	0.11
Fee charge (in LAK)	53 308 (105 995)	43 166 (89 158)	0.74

* LAK = Lao KIP; 1 USD = 8 500 LAK

There were no differences regarding quality of treatment for patients at different income levels as measured through the scores for appropriate drug use (5.0, 4.8 and 4.8, for low-, medium- and high-level income respectively, *p *= 0.71). Furthermore, there were no significant differences in how patients with different levels of income were treated regarding use of medicines, for example, average number of antibiotic injections (0.87, 0.93 and 0.94 for low-, medium- and high-level income respectively, *p *= 0.63), and total cost of medicines (LAK 319 645, 324 685, and 370 855 respectively, *p *= 0.85). However, there was a significant difference regarding fees, where patients in the high-level income group paid about three times more than the two other income groups (*p *< 0.0001).

The total level of the score for appropriate use of medicines was significantly lower for patients with major injury and post-delivery haemorrhage or a Caesarean section (3.7 and 3.8, respectively), than for patients with hypertension (5.8) and for children with pneumonia (7.1) (*p *< 0.0001). The low score for patients with major injury and post-partum treatment were mainly due to incorrect choice of intravenous fluids and inappropriate use of oral antibiotics. The average use of antibiotic injection was 1.0 in patients with major injury, 1.5 in patients with post-delivery haemorrhage or a Caesarean section, 0.04 in hypertensive patients, and 1.1 in children under five with pneumonia, which for all tracers mostly corresponded with recommendations in the guidelines. The cost for patients with major injury and post-delivery haemorrhage or Caesarean section was significantly higher than for the other two conditions (*p *< 0.0001).

Patients who made informal payments had a significantly lower score for appropriate use of medicines than those not making informal payments (Table 3). The prescribing of medicines and the number of days in hospital were significantly higher for patients making informal payments than for those not making informal payments, and they had to pay significantly more for medicines and diagnostic tests than those not making informal payments. The proportion of patients who paid informally was 18%, 16% and 8.9% for the high, middle and low level of incomes respectively. Only nine out of the total of 110 patients with informal payment were covered by insurance. The informal payment was mostly in cash (82%) and to a lesser extent in kind (18%), e.g., fruit, soft drinks and meals.

**Table 3:** Use of medicines, diagnostic test and cost for patients who made and did not make informal payment respectively

Descriptions	Means (SD)		p-value
		
	No informal payment N = 674	Informal payment N = 110	
Appropriate drug use score	5.1(2.7)	3.5(2.7)	<0.0001
Total number of medicines prescribed	5.3(2.6)	6.7(2.2)	<0.0001
- Number of antibiotic injections	0.80(0.88)	1.4(0.89)	<0.0001
- Number of oral antibiotics	0.49(0.61)	0.30(0.46)	0.001
- Number of essential medicines	4.6(2.2)	5.4(1.9)	<0.0001
- Number of brand drugs	1.3(1.4)	1.4(1.7)	0.78
Average duration of stay (days)	3.2(3.9)	6.8(5.6)	<0.0001
Total diagnostic test	1.2(1.5)	2.2(1.3)	<0.0001
Cost of medicines	245 087 (560 887)	1 058 000 (1 236 000)	<0.0001
Cost of diagnostic test	65 117 (103 274)	119 906 (162 750)	<0.0001
Other costs	285 518 (497 730)	688 615 (678 346)	<0.0001
Fee charge	52 255 (102 529)	69 731 (130 124)	0.52

The score for appropriate use of medicines was significantly higher at district hospitals than at provincial hospitals (5.3 vs. 4.8, *p *= 0.01). The use of medicines for patients at provincial level was also significantly higher than for patients at district level in terms of average number of medicines prescribed (6.8 vs. 4.5, *p *< 0.0001), number of antibiotics injections (1.3 vs. 0.7, *p *< 0.0001) and number of essential medicines (5.7 vs.3.9, *p *< 0.0001), as well as number of days in hospital (6.6 vs.1.7, *p *< 0.0001). Furthermore, the patients' expenditure for medicines (LAK 740 350 vs. 113 100, *p *< 0.0001) and diagnostic tests (LAK 95 478 vs. 47 023, *p *< 0.0001) was significantly higher at provincial level although the same tracer conditions were used at both levels.

There were also some differences between the provinces. Patients in Vientiane province received significantly fewer medicines (4.2 vs. 5.7 and 5.8, *p *< 0.0001) and antibiotics injections (0.5 vs. 1.1 and 1.3, *p *< 0.0001), and fewer diagnostic tests (0.4 vs. 1.3 and 1.9, *p *< 0.0001) than patients in the two other provinces. Patients in Savannakhet province stayed longer in hospital (8.9 vs. 3.8 and 0.8, *p *< 0.0001) than in the two other provinces and they also spent more on medicines (LAK 1 218 000 vs. 316 322 and 109 800, *p *< 0.0001), diagnostic tests (LAK 120 782 vs. 57 838 and 77 071, *p *= 0.009) and non-medical expenditures (LAK 730 471 vs. 131 540 and 110 129, *p *< 0.0001).

A higher total cost for medicines and diagnostic tests was associated with uninsured patients, patients with higher education, patients making informal payments, patients treated in a provincial hospital and patients in the lowest income-level group (Table 4). There was no association found with sex, profession and distance from health facility.

**Table 4:** Suggested factors influencing provider performance in terms of total cost for medicines and diagnostic tests in LAK*, multiple linear regression analysis (R-squared = 0.29).

Suggested factors	beta coefficient	Standard deviation	*p*-value	95% confidence limits
***Sex*** 0 = female, 1 = male	-58 571	50 455	0.246	(-157 625; 40 482)
***Education*** 0 = low, 1 = high	117 142	59 380	0.049	(568; 233 715)
***Income*** 0 = low, 1 = high	-139 999	52 653	0.008	(-243 366; -36 632)
***Hospital level*** 0 = provincial, 1 = district	-578 531	52 880	<0.001	(-682 344; -474 717)
***Insurance*** 0 = no insurance, 1 = insurance	-178 853	71 359	0.012	(-318 943; -38 763)
***Informal payment*** 0 = no informal payment 1 = with informal payment	681 790	74 142	<0.001	(536 236; - 827 344)

* LAK = Lao Kip; 1 USD = 8 500 LAK

## Discussion

Prescribing practices were significantly more appropriate for insured patients, and for patients not making informal payments. In addition, the total cost of medicines and diagnostic tests was as well significantly lower for insured patients and for patients not making informal payments. However, there was no difference between poor and better-off patients. Factors associated with the cost of medicines and diagnostic tests, which may have influenced prescribing and treatment practices, were insurance schemes, education, low-income level patients, level of health facility, and informal payment.

Insured patients received significantly more appropriate medicines and diagnostic tests than those without insurance. The different insurance schemes in Laos apply their own lists of medicines, but mostly follow the National Essential Medicine List with generic names. Providers were encouraged to treat insured patients according to the list and the insured patients also received fewer drugs, fewer antibiotics, fewer injections in total and the expenditure were lower. Uninsured patients, on the other hand, could receive medicines at the prescriber's discretion and according to the patient's own demands. These different preconditions seem to have had an impact on the doctors' prescribing habits. A study in China showed that insured patients instead received more antibiotics and injections than needed possibly because prescribers received a bonus from pharmacies from over-prescribing, and because the income of health workers was directly related to the drugs they sold [3,15], which is not the case for insured patients in Laos. Studies in other countries have also addressed the issues of unethical practices at the cost of consumers [2,4,5].

Although the use of antibiotics was less for insured patients and patients not making informal payments, than for uninsured patients and those making informal payments, on average, each patient still received at least one antibiotic, except for patients with hypertension. This was regarded as appropriate in many cases as children with pneumonia and patients with wound injuries should be treated with antibiotics. Appropriate use of antibiotics is important to avoid antibiotic resistance, as bacteria resistance is currently on the increase world-wide, while the development of new antibiotic development is in decline [16,17]. In a three-country comparison, the level of microbial resistance was highest in China, followed by Kuwait and the United States [18]. Other studies in China have shown that antibiotic prescribing and use was connected with the interaction between physicians and patients [15,19]. A study in Pakistan and other studies have also demonstrated irrational prescribing of injections and antibiotics [3,20,21].

Patients with major injury or bone fracture and post-delivery haemorrhage or a Caesarean section received less appropriate overall treatment than patients with hypertension or pneumonia. We cannot fully explain the reasons for this discrepancy, but it may partly be related to the diversity of conditions covered by the guidelines for non-infectious emergency cases.

We found that patients with a lower income level received similar treatment as the middle- and higher-level income groups, although patients in the highest income-level group had higher total expenditure. In each hospital, an exemption policy was principally implemented: if patients could not pay, they were first allowed credit, and then their debts were cancelled if it turned out that they really were unable to pay. In Laos, rational use of medicines has been promoted for over fifteen years and Drug and Therapeutic Committees have been established and function at all provincial and several district hospitals [9,10]. Therefore, patients should get the same treatment regardless of level of income, which is consistent with our findings and similar to other studies, where it has been shown that providers generally have a positive attitude towards deprived patients [22].

Patients making informal payments, who were also mostly non-insured, received inappropriate treatment, to a higher extent than patients not making informal payments. One possible reason could be that prescribers tried to satisfy explicit or assumed demands from non-insured patients by over-prescribing or choosing famous brand name drugs at a substantially higher cost to the patient. A study in Russia showed that in total around 15% of respondents had made informal payments in the past three years, although it was even more common in poorer regions. The reason for payment was said to be to ensure access to high quality, publicly funded health care services [6]. In a study in Greece, 36% of the total number of those reporting treatment at public hospitals reported at least one informal payment to a doctor. The reasons for payment included a fear of receiving substandard care, an attempt to shorten waiting time and in some cases because the doctor had demanded such a payment [7].

Patients at district hospitals spent less money on treatment than those at provincial hospitals for the same tracer conditions. The explanation could be that health staff at district level provided less advanced care, although the appropriateness of the care was significantly higher. However, even if the condition is classified within the same tracer, the cases at the provincial hospitals may have been more severe.

Study limitations included the much lower number of insured than uninsured patients due to the recent, incomplete introduction of insurance schemes. For the sake of comparison, it would have been an advantage to have more equal groups. The income level reported by the patients may not be appropriate, which may affect the comparisons between groups of patients with different income levels.

## Conclusions

Use of medicines and cost of care at hospitals in Laos were more appropriate for insured patients regardless of level of income. The lower cost for insured patients can help reduce the number of households experiencing catastrophic health expenditure. The positive effects of insurance schemes on expenditure and appropriate use of medicines may be associated with long-term effects of promoting rational use of drugs, including support to active DTC work. The Government should continue to support the scaling-up and strengthening of social security schemes and to implement a policy to prohibit informal payment. Factors related to higher cost and lower quality of care for patients who make informal payments should be further explored.

## Competing interests

The authors declare that they have no competing interests.

## Authors' contributions

LS developed and implemented the study, analysed and interpreted the data and wrote the manuscript; DS participated in design, data collection, and interpretation of findings and commented on the manuscript; GT participated in design and interpretation of findings, and commented on the manuscript; MP participated in design, regression analysis and interpretation of findings, and commented on the manuscript; CR participated in design, interpretation of findings and commented on the manuscript; RW participated in design, supported the implementation, data analysis and interpretation of findings, and commented on the manuscript. All authors have read and approved the final manuscript.

## Pre-publication history

The pre-publication history for this paper can be accessed here:

http://www.biomedcentral.com/1472-6963/11/3/prepub
